# Rapid effect of benralizumab in exacerbation of severe eosinophilic asthma associated with eosinophilic granulomatosis with polyangiitis

**DOI:** 10.1186/s12890-021-01397-7

**Published:** 2021-01-21

**Authors:** Carlos Martínez-Rivera, Ignasi Garcia-Olivé, Blanca Urrutia-Royo, Maria Basagaña-Torrento, Antoni Rosell, Jorge Abad

**Affiliations:** 1 Pneumology Department, Hospital Universitari Germans Trias I Pujol, CIBERES, Universitat Autònoma de Barcelona, Carretera del canyet sn, 08916 Badalona, Barcelona Spain; 2 Allergy Department, Hospital Universitari Germans Trias I Pujol, Badalona, Spain

**Keywords:** Severe eosinophilic asthma, Benralizumab, Eosinophilic granulomatosis with polyangiitis, Pulmonary function

## Abstract

**Background:**

Eosinophilic granulomatosis with polyangiitis (EGPA) is a disease that is associated with severe uncontrolled eosinophilic asthma. Eosinophils play an important pathogenic role in the development of both diseases. Benralizumab is an antieosinophilic monoclonal antibody that binds to the α subunit of the human interleukin 5 receptor that is expressed on the surface of the eosinophil and basophil. We present the first case of rapid improvement in symptoms and lung function during admission for exacerbation of a severe eosinophilic asthma associated with EGPA.

**Case presentation:**

A 57-year-old man diagnosed with severe eosinophilic asthma associated to EGPA was admitted to the Pulmonology Department due to severe bronchospasm. At admission he presented 2300 eosinophils/µl. Despite intensive bronchodilator treatment, intravenous methylprednisolone at a dose of 80 mg/d, oxygen therapy, and budesonide nebulization, the patient continued to present daily episodes of bronchospasm. Ten days after admission, with blood eosinophil levels of 1700 cells/µl, benralizumab 30 mg sc was administered. That day, the Forced Expiratory Volume in the first second (FEV1) was 28% of the theoretical value (1150 ml). AT three days, FEV1 increased to 110 ml (31%). On the 9th day FEV1 was 51% (2100 ml). The blood eosinophil level on the 9th day was 0 cells/µl.

**Conclusions:**

The rapid improvement of FEV1 is in line with studies based on clinical trials that found improvement after two days in peak flow and one phase II study that showed rapid response in exacerbation of asthma in the emergency room. The antieosinophilic effect at 24 h and the effect in different tissues determine the rapid improvement and the potential advantage of benralizumab in the treatment of EGPA. This case suggests the usefulness of benralizumab in patients with EGPA and eosinophilic severe asthma who show bronchospasm refractory to conventional treatment during a hospitalization due to asthma exacerbation.

## Background

Eosinophilic granulomatosis with polyangiitis (EGPA) is a disease that causes small to medium sized vessel necrotizing vasculitis and affects many organs to varying degrees, including the lungs, and is associated with severe uncontrolled eosinophilic asthma. Eosinophils play an important role in the development of both pathologies. The pathogenesis of EGPA is not well known. It is believed to be the product of the interaction of genetic factors and exposure to environmental factors such as infectious agents, birds, cocaine or medicines*.* As a result an inflammatory response is unleashed involving eosinophils and where it is believed that the clonal expansion of TCD8 cells and to a lesser degree TCD4 cells can play a role [1].

Benralizumab is an antieosinophilic monoclonal antibody that binds to the α subunit of the human interleukin 5 receptor (IL-5Rα) that is expressed on the surface of the eosinophil and basophil. Through an antibody-dependent cellular cytotoxicity mechanism and because of its high affinity for FcγRIII receptors in immune effector cells such as natural killer (NK) cells, benralizumab produces apoptosis of eosinophils [2]. The direct action mechanism of benralizumab on the eosinophils involved in systemic inflammation [3] suggests that it could be effective in improving asthma in patients with EGPA.

It is known that benralizumab can produce rapid improvement in lung function (8) and that it may be useful for treating EGPA (11), in stable situations. But there is no evidence on the efficacy in these diseases during an exacerbation. We report a case of a patient admitted to the hospital for exacerbation of a Severe Eosinophilic Asthma associated with EGPA refractory to conventional treatment, who presented significant improvement in symptoms and lung function two days after starting the administration of benralizumab during hospitalization.

## Case presentation

Our patient is a 57-year-old male diagnosed with asthma 4 years ago in another center and treated with a combination of a high-dose inhaled corticosteroid (budesonide 1280 µg/d) and a long-acting β2-adrenergic agonist, formoterol 36 µg/d (ICS/LABA) and a long-acting antimuscarinic (LAMA), tiotropium 5 µg/d. He did not report daily symptoms but required treatment with courses of oral corticosteroids 3–4 times annually for the last 4 years. Six months before admission he had Forced Expiratory Volume in the first second (FEV1) of 58% (2388 ml).

Three months prior to the presentation discussed the patient was diagnosed of EGPA with pulmonary involvement in the form of multiple bilateral ground glass infiltrates, chronic pansinusitis and cutaneous vasculitis in the extremities and buttocks, diagnosis by cutaneous biopsy showing fibrinoid necrosis and perivascular eosinophilic infiltrate. He had high levels of p antineutrophil cytoplasmic antibody (pANCA)(1). Historically, the maximum levels of eosinophils in the blood have been 20,000 cells/µl. Upon admission, he presented 2300 cells/µl. One month earlier, the patient was admitted to the Rheumatology Department of our center and required treatment with 3 boluses of 500 mg of methylprednisolone. He had been on prednisone 60 mg/d from then until the current admission.

The patient was admitted to the Pulmonology Service due to severe Bronchospasm with respiratory rate of 35 rpm, bilateral hypophonesis and respiratory failure (pH: 7.36; pCO_2_: 51 mmHg; pO2: 51 mmHg; HCO_3_: 28.8 mmol/L; Sat. O_2_: 88.6%). His general condition was poor, and he presented dyspnea with inability to the supine. Intensive bronchodilator treatment and systemic corticosteroids were started, with which the patient improved rapidly. After a 6 h stay in the emergency dept., admission to the conventional ward was decided since he was stable with some persistent scattered wheezing, with O_2_ Saturation of 96% under O_2_ venturi typo oxygen mask delivering 28% oxygen.

Despite bronchodilator treatment, intravenous methylprednisolone at a dose of 80 mg /d, oxygen therapy, and budesonide nebulization, the patient continued to present daily episodes of bronchospasm accompanied by a refractory cough during the first 10 days of admission. We assumed that persistent eosinophilic inflammation despite corticosteroid treatment played an important role in his incomplete recovery, thus proposing a rapid eosinophilic suppression pharmacotherapeutic alternative.

Ten days after admission, with blood eosinophil levels of 1700 cells/µl, benralizumab 30 mg sc was administered. Spirometry that day showed, prior to the start of treatment, a FEV1 of 28% of the theoretical value (1150 ml).

Within three days of starting benralizumab treatment, the patient had less dyspnea, the cough improved significantly, as well as his condition. FEV1 showed increases of 110 ml (31% theoretical value). On the sixth day after starting benralizumab, the FEV1 improved to 45% (1830 ml) of the theoretical value and there were no new episodes of dyspnea plus wheezing. On the 9th day the clinical improvement persisted and the FEV1 was 51% of the theoretical one (2100 ml), representing an improvement of 985 ml compared to the first spirometry. At that time, he had a blood eosinophil level of 0 cells/µl. Ten days after starting benralizumab, the patient was discharged and ordered to gradually decrease the treatment with oral corticosteroids in the next 20 days until maintaining 5 mg/d of prednisone. No adverse effects were reported. The patient explained that, from his point of view, he had never experienced such a clear improvement as now.

## Discussion and conclusions

**Fig. 1:**
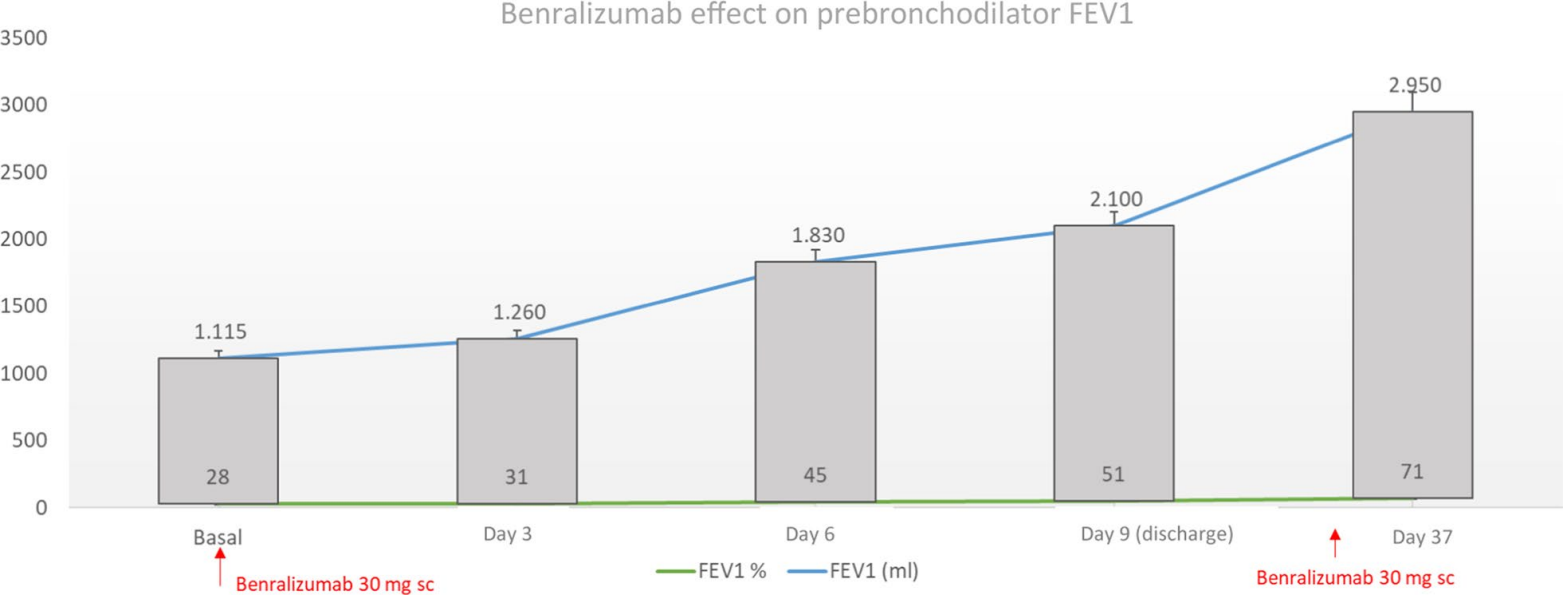
Evolution of lung function according to prebronchodilator FEV1 (% theoretical value and ml) after the first dose of benralizumab 30 mg sc and after two days following the second dose

The rapid improvement in lung function in this patient from a baseline prebronchodilator FEV1 value of 1150 ml was maintained after discharge. One month after starting treatment and three days after the administration of the second dose of benralizumab, a FEV1 of 2950 ml was reached (71% of the theoretical value) (Fig. 1). In line with these data, a study based on three clinical trials reported improvement after two days in peak flow [6]. This increase was accompanied by a significant improvement in forced vital capacity (FVC) that increased by 1250 ml from baseline, which illustrates the impact that benralizumab causes on lung hyperinflation. High levels of eosinophils in the blood are associated with airway limitations [7] and it seems likely that the antieosinophilic effect at 24 h of this monoclonal antibody may be related to the rapid increase in respiratory function parameters, as described by pivotal studies [8] and confirmed in real life studies with benralizumab [9]. On the other hand, in a phase II trial, eligible subjects in the emergency room with an exacerbation of asthma had a rapid response with benralizumab, with the hypothesis that it may act as an adjunct treatment to oral corticosteroids in the treatment of exacerbations and prevent recurrence of the same [4].

Likewise, the effect on eosinophils in different tissues involved in the inflammatory response determine a potential advantage of benralizumab in the treatment of EGPA. Studies are being carried out that will explore the efficacy and safety of benralizumab in the treatment of this condition [10] and that will validate the results experienced in some patients [5, 11].

Our case shows a rapid response in both symptoms and lung function in a patient with exacerbated severe eosinophilic bronchial asthma and EGPA, associated with a total depletion of eosinophils in the blood a few days after starting treatment. This suggests that in patients with this profile who show a bronchospasm refractory to conventional treatment during admission, the start of treatment with benralizumab could be a therapeutic option to consider.

## Data Availability

The datasets used and/or analyzed during the current study are available from the corresponding author on reasonable request.

## Abbreviations

EGPA: Eosinophilic granulomatosis with polyangiitis
IL-5Rα: α Subunit of the human interleukin 5 receptor
NK: Natural killer
ICS: Inhaled corticosteroid
LABA: Long-acting β2-adrenergic agonist
LAMA: Long-acting antimuscarinic
pANCA: P antineutrophil cytoplasmic antibody
FEV1: Forced expiratory volume in the first second
